# The Maltase Involved in Starch Metabolism in Barley Endosperm Is Encoded by a Single Gene

**DOI:** 10.1371/journal.pone.0151642

**Published:** 2016-03-24

**Authors:** Vasilios M. E. Andriotis, Gerhard Saalbach, Robbie Waugh, Robert A. Field, Alison M. Smith

**Affiliations:** 1 John Innes Centre, Norwich Research Park, Norwich, United Kingdom; 2 James Hutton Institute, Invergowrie, United Kingdom; University of Potsdam, GERMANY

## Abstract

During germination and early seedling growth of barley (*Hordeum vulgare*), maltase is responsible for the conversion of maltose produced by starch degradation in the endosperm to glucose for seedling growth. Despite the potential relevance of this enzyme for malting and the production of alcoholic beverages, neither the nature nor the role of maltase is fully understood. Although only one gene encoding maltase has been identified with certainty, there is evidence for the existence of other genes and for multiple forms of the enzyme. It has been proposed that maltase may be involved directly in starch granule degradation as well as in maltose hydrolysis. The aim of our work was to discover the nature of maltase in barley endosperm. We used ion exchange chromatography to fractionate maltase activity from endosperm of young seedlings, and we partially purified activity for protein identification. We compared maltase activity in wild-type barley and transgenic lines with reduced expression of the previously-characterised maltase gene *Agl97*, and we used genomic and transcriptomic information to search for further maltase genes. We show that all of the maltase activity in the barley endosperm can be accounted for by a single gene, *Agl97*. Multiple forms of the enzyme most likely arise from proteolysis and other post-translational modifications.

## Introduction

Starch degradation in the endosperm of barley seedlings provides the glucose substrate for the early growth of the seedling. Conversion of starch polymers to glucose involves four classes of enzyme, α-amylase, β-amylase, limit-dextrinase and maltase. While the first three of these classes are well understood at the gene, transcriptional and protein level in the endosperm [1–9] and in the context of brewing and distilling [10–13], relatively little is known about maltase. Its nature, its role in endosperm starch degradation and its importance for brewing and distilling remain subjects of debate.

Maltases are specialised α-glucosidases (exo-acting enzymes that release α-d-glucose from the non-reducing end of their substrates) that act preferentially on maltose, producing glucose. Maltases are also able to release α-d-glucose from the non-reducing end of generic α-glucosidase substrates such as 4-nitrophenyl α-D-glucopyranoside (pNPG), but at rates lower than that for maltose hydrolysis [14]. It has long been known that germinating barley grains contain one or more enzymes capable of the hydrolysis of maltose [15]. At least some of this maltase activity is attributable to Agl97, an α-glucosidase of glycoside hydrolase family GH31 with a high affinity for maltose [16–19]. There is evidence for the presence of other maltases in the endosperm. Fractionation by ion exchange chromatography of soluble proteins from germinating grains consistently revealed the presence of at least two separable maltase activities [20–23]. Agl97 encodes a protein with a molecular mass of 97 kDa [16,19] predicted to be processed by removal of an N-terminal signal peptide to a mature mass of 92 kDa. Although a protein of this mass was purified from barley endosperm by Frandsen and colleagues [18], maltases with masses of 66 and 14 kDa [20], and 33 kDa [23] have also been partially purified. Henson and colleagues reported the occurrence in barley of four distinct genes encoding α-glucosidases (named Agl1 to Agl4 [24] see http://www.cazy.org/GH31), although it is not clear whether any of the gene products preferentially hydrolyse maltose. Thus the number and nature of maltases in the endosperm of barley seedlings remains to be determined.

The role of maltases in the endosperm of barley seedlings is also poorly understood. Sun and Henson reported from in vitro experiments that two maltase activities purified from barley endosperm could hydrolyse soluble and granular starch as well as maltose in vitro, and that these enzymes strongly and synergistically promoted the degradation of granules in the presence of α-amylases [21,25]. A subsequent study [23] failed to reproduce these strong synergistic effects, and the authors proposed that previous maltase preparations [21] were contaminated with other hydrolytic enzymes.

The quality of barley malt–the substrate for the brewing and distilling industries–is largely determined by the activities in the endosperm of enzymes of starch degradation. During the malting process, barley grains are hydrated and then allowed to germinate, typically over three to four days. Maltsters aim to maximise the activities of enzymes while minimising the loss of starch and the growth of the embryo. The barley amylases are crucial for conversion of starch to sugars in the subsequent mashing stage of alcohol production, but maltase is heat labile and much of it may be denatured at the high temperatures imposed during mashing [26,27]. Some authorities argue that introduction of a thermostable maltase would reduce potential inhibition of amylases by maltose and–if maltases can indeed synergistically promote starch granule degradation by amylases—could potentially increase the rate of conversion of starch to fermentable sugars [21,27–29]. However, others propose that the presence of maltase is detrimental during the mashing stage of brewing—in part because the resulting elevated glucose levels can potentially inhibit maltose uptake and lead to over-production of flavour compounds by brewer’s yeast [26,30,31]. Full evaluation of the potential for improving malt quality by manipulation of maltases thus requires more information on the nature and roles of the endogenous maltases [12].

We previously used transgenic and chemical approaches to shed further light on the maltases of barley endosperm [32]. We showed that seedlings of lines carrying an RNA interference silencing cassette for *HvAgl97* had reductions of up to 50% in α-glucosidase activity (measured with the generic α-glucosidase substrate pNPG) in the endosperm. The ratio of glucose to maltose in the endosperm of seedlings was strongly reduced in these lines, but the patterns of starch degradation and seedling growth were unaffected. Different effects were seen when wild-type seedlings were grown in the presence of deoxynojirimicin (DNJ), an established inhibitor of α-glucosidases [33] that has been shown to inhibit Agl97 [32]. The glucose to maltose ratio was strongly reduced as in the transgenic lines, but both starch degradation and seedling growth were also inhibited.

These results suggested first that Agl97 is important for conversion of maltose to glucose but may play no role in degradation of starch granules in vivo, and second that the endosperm of barley seedlings contains DNJ-sensitive enzymes other than Agl97 that are required for normal rates of starch degradation. Since DNJ does not inhibit amylases [32], we speculated that its effects on starch degradation are either via inhibition of unidentified enzymes that act on starch granules, or via inhibition of other unknown enzymes indirectly required for normal starch degradation [32].

In this paper we investigate the possibility that the barley endosperm contains unidentified maltases that may be necessary for normal rates of starch degradation. We use protein fractionation and identification techniques on wild-type seedlings and seedlings of transgenic lines with reduced expression of Agl97 to investigate the extent to which Agl97 accounts for the maltase activity in the endosperm.

## Results

**Fig 1:**
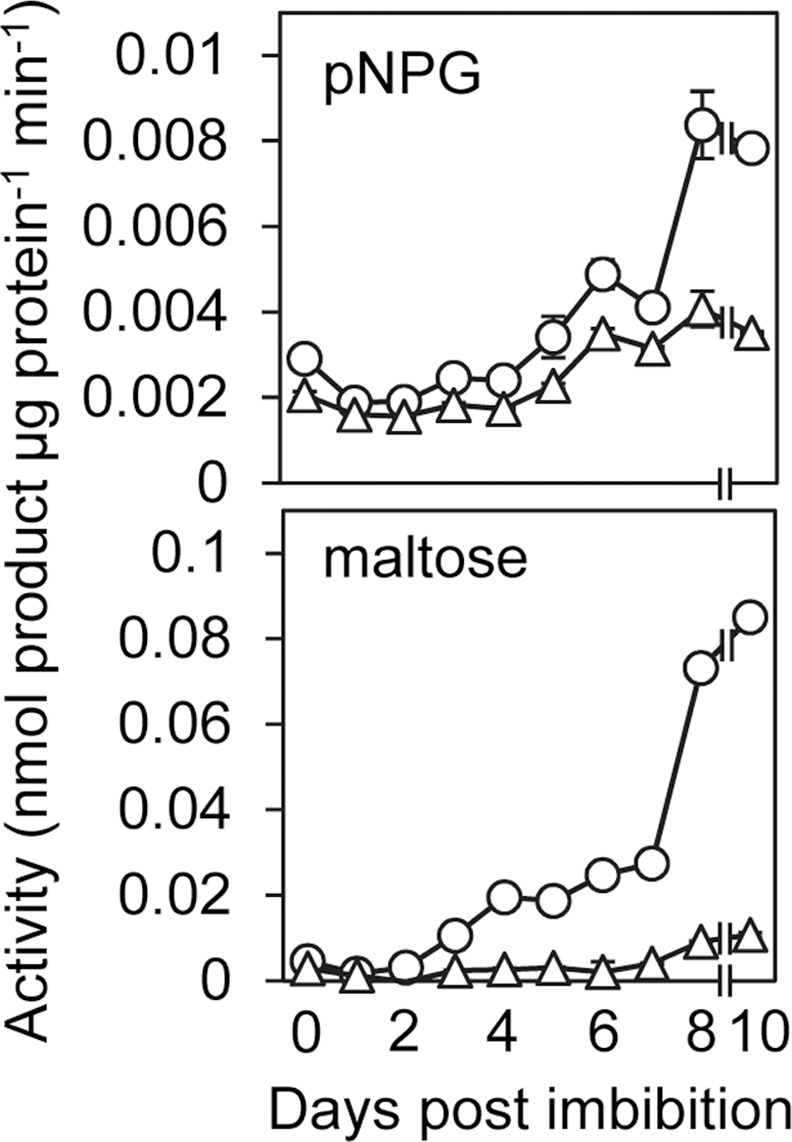
Time course of development of maltase and α-glucosidase activity in the endosperm. Extracts were prepared from endosperm pooled from five seedlings at each time point. Values are means of three technical replicates ± SE. Circles, without inhibitor; triangles, with 0.5 mM DNJ in the assay. Upper graph, assay with pNPG as the substrate. Lower graph, assay with maltose as the substrate.

To determine the optimal time at which to study maltase activity, we assayed maltase and general α-glucosidase activity (using maltose and the generic α-glucosidase substrate pNPG, respectively) during the first ten days post imbibition (dpi) of barley grains (cv NFC Tipple). Both activities increased sharply in the endosperm from about 3 dpi (Fig 1). Whereas maltase activity was undetectable prior to this point and was almost completely inhibited by inclusion of 0.5 mM DNJ in the assay, α-glucosidase activity was present from the point of imbibition. This early activity was not inhibited by DNJ, but it was increasingly susceptible to DNJ inhibition from about six days onwards (Fig 1). Based on these results, we performed subsequent experiments on barley endosperm at 10 dpi. At this point the α-glucosidase and the maltase activities are maximal, more than 75% of the starch originally present in the grain has been degraded, and levels of both maltose and glucose in the endosperm have peaked ([32], and our observations).

**Fig 2:**
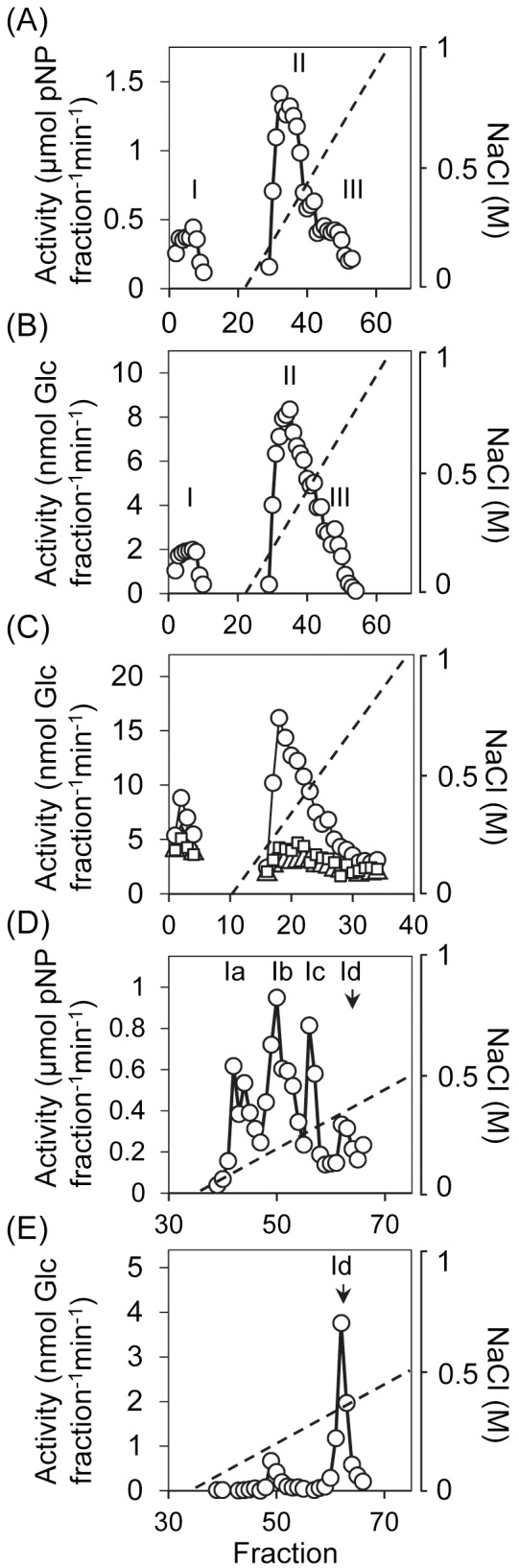
Fractionation on a MonoQ anion exchange column of maltase activity from endosperm. All fractionations were reproduced at least twice, on independent batches of seedlings. Representative examples are shown. (A), (B). Extracts of endosperm of seedlings of cv NFC Tipple at 10 dpi were applied to a MonoQ column by FPLC. Proteins binding the column were eluted with a gradient of increasing NaCl concentration (dashed line). One-mL fractions were collected and assayed for α-glucosidase activity with pNPG (A), or maltase activity with maltose (B). I-III indicate the three peaks of activity. (C). Extracts were prepared, fractionated and assayed for maltase as in (B), except that seedlings were either transgenic lines in a cv Golden Promise background carrying an RNA interference (RNAi) silencing cassette for *Agl97* [lines 21 and 23 [31] (squares)], or an out-segregant line (i.e. not carrying the silencing cassette) from the same transformation experiment (circles). Chromatographic conditions were the same as in (B) except that the column wash to remove unbound proteins was reduced from 20 to 10 mL. Note that peak II maltase activity elutes at the same NaCl concentration in the experiments shown in (B) and (C). (D), (E). Fractionation on a MonoS column of proteins not binding to a MonoQ column [peak I in (A) and (B)]. Proteins binding to the MonoS column were eluted with a gradient of increasing NaCl concentration (dashed line). One-mL fractions were collected and assayed for α-glucosidase activity with pNPG (D), or for maltase activity with maltose (E). Peaks of activity eluting from the column are designated Ia-Id.

To identify maltases present in the endosperm, proteins that precipitated from endosperm extracts between 20% and 80% (w/v) ammonium sulfate (a fraction that contained essentially all of the maltase activity) were fractionated on a MonoQ anion exchange column. Under our chromatographic conditions, α-glucosidase and maltase activities had very similar profiles. Both appeared in the unbound fraction (peak I) and during elution of bound proteins with an increasing NaCl gradient (peaks II and III: Fig 2A and 2B).

To discover which peak(s) are attributable to the previously-identified maltase Agl97, we repeated these experiments with extracts from endosperm of transgenic lines of barley (cv Golden Promise) carrying an RNA interference (RNAi) silencing cassette for *Agl97* (lines 21 and 23 [32]). These transgenic lines carry a single insert of the transgene. Relative to an out-segregant control line they have greatly reduced α-glucosidase activity but normal rates of starch degradation in the endosperm and normal seedling growth [32]. The elution profile on a MonoQ column of maltase activity from endosperm of the out-segregant control line closely resembled that of extracts from barley cv NFC Tipple. However, in extracts from the transgenic lines 21 and 23 the activity in all peaks (I-III) was strongly reduced (Fig 2C). This result indicates that maltase activity in the endosperm is largely attributable to Agl97, and/or to enzyme(s) encoded by genes highly similar to *Agl97* and thus susceptible to silencing by the RNAi cassette.

To fractionate the unbound activity in peak I, this material was applied to a MonoS cation exchange column at pH 5. Under these conditions, the α-glucosidase activity bound to the column and eluted in four distinct peaks at 150, 250, 350 and 400 mM NaCl [peaks Ia, Ib, Ic and Id respectively (Fig 2D)]. By contrast, maltase activity eluted as a single peak that corresponded to peak Id of α-glucosidase activity (Fig 2E). Maltase activity in peak Id was purified 170-fold relative to that in crude extracts of endosperm, with a recovery of ~10% (Table 1).

**Table 1:** Purification of maltase from endosperm extracts.

	Protein	Specific activity	Total activity	Recovery	Fold purification
	(mg)	(nmol μg^-1^ min^-1^)	(μmol min^-1^)	(%)	
Crude	45	0.1	3.6	100	1
20–80% ammonium sulfate	30	0.1	4	111	1.7
MonoQ (unbound)	6	0.4	2	56	4.5
MonoS, peak Id	0.025	13.5	0.34	9.5	168

Recovery is the percentage of the initial (crude extract) activity remaining at each step of the purification. Fold purification is the fold increase in specific activity relative to the crude extract at each stage of the purification.

MALDI-ToF mass spectrometry was used to identify proteins in peak Id (S1 Table). Tryptic digestion of proteins in peak Id prior to fractionation on SDS-PAGE yielded 13 unique peptides that matched with the following known or predicted α-glucosidases: Agl97 (Q9LLY2 [16,18], coverage of 24%), an α-glucosidase identical to Agl97 (D1MDV2, Agl1), and a predicted protein essentially identical to Agl97 except for an additional 53 amino acids N-terminal to the Agl97 sequence (F2DV72; predicted from mRNA AK367790.1). This N-terminal peptide is not predicted to be a secretion or transit peptide, or to have a cleavage site (PSORT, SignalP; http://expasy.org/resources/). It is not found in predicted Arabidopsis or rice proteins that are most similar to Agl97 (At5g11720 and Os06g46284 respectively; see https://ics.hutton.ac.uk/morexGenes/view_gene2.cgi?seq_name=MLOC_66806&dataset=assembly3_WGSMorex_rbca.fasta#homologies). Searches of translated nucleotide databases with the 53 amino acid peptide did not identify other plant glycosyl hydrolases containing this sequence.

**Fig 3:**
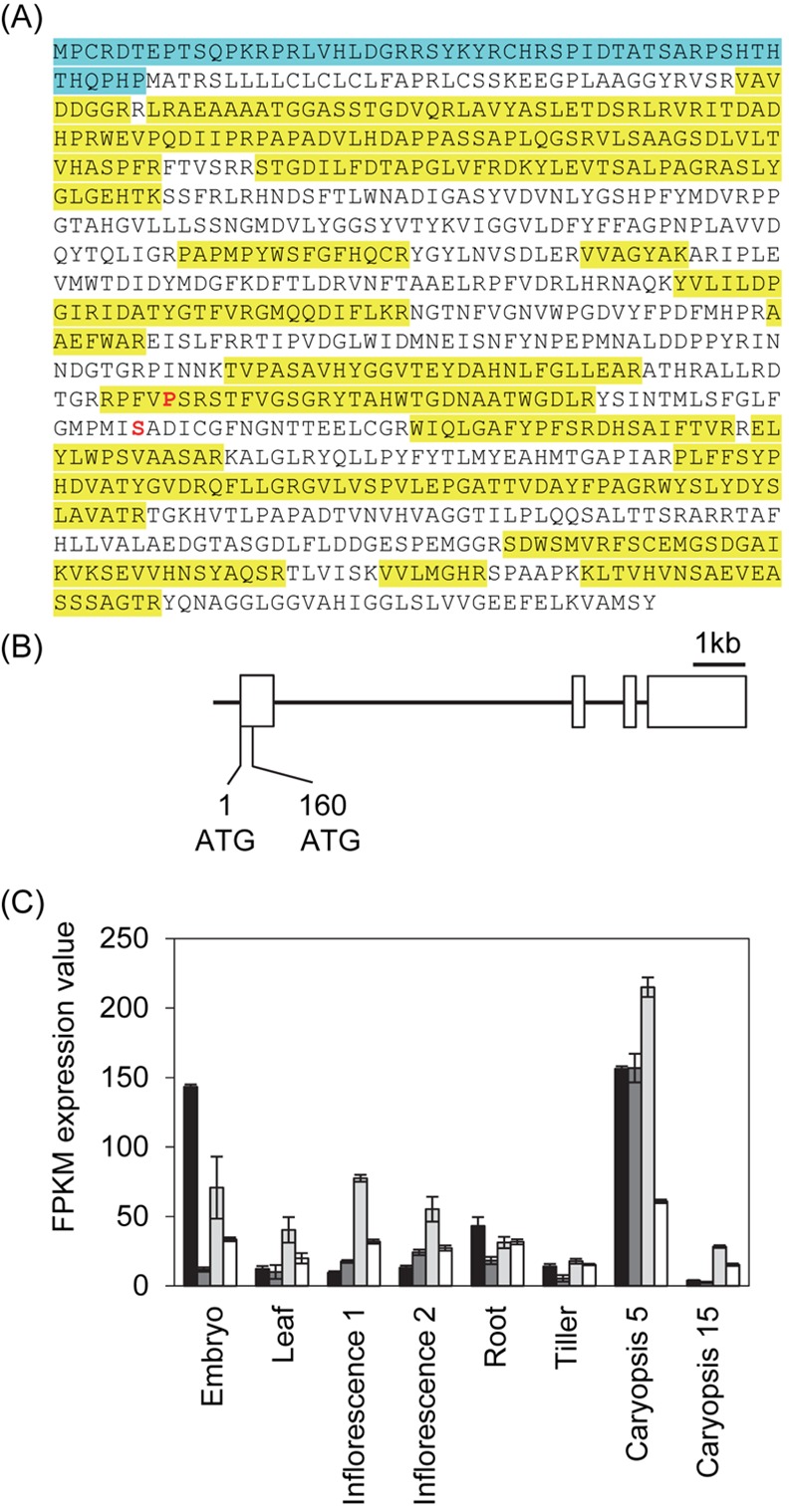
Identification of Maltase-Like Proteins Following Purification, and Structure of the Gene Encoding Them. (A) Predicted amino acid sequence of maltase-like proteins Agl97 (Q9LLY2), Agl1 (D1MDV2) and predicted protein (F2DV72), and coverage of the sequence by tryptic peptides identified by mass spectrometry in peak Id from Fig 2E. These three separately-annotated barley proteins have near-identical predicted amino acid sequences. Agl97 and Agl1 are identical, whereas the predicted protein F2DV72 has an additional 53 amino acids at the N-terminus (blue), and also differs with respect to the P and S residues shown in red: in Agl1 and Agl97 these are respectively L and G. Sequences in yellow were identified among tryptic peptides from peak Id of maltase activity recovered after purification. A full list of the maltase peptides identified is in S1 Table. (B) Structure of the gene encoding the three maltase-like proteins. Boxes represent exons. The first exon encodes the N-terminal amino acid sequence unique to F2DV72 (from bp 1 to bp 159), and a further 73 amino acids (from an ATG at bp 160) that are common to all three predicted maltase-like proteins. The remaining three exons encode amino acid sequences common to the three proteins encoded by the remaining three exons. (C) Expression pattern of the *HvAgl* genes. Results are average values from three biological replicates per tissue (± SE) and were obtained from the International Barley Genome Sequencing Consortium [34]. Data are in FPKM expression values (fragments per kilobase of exon per million fragments mapped). Black bars: *Agl1/Agl97*; grey bars: *Agl2*; light grey bars: *Agl3*, open bars: *Agl4*. Tissues analysed were: embryo (from germinating grains at 4 dpi, including root, coleoptile and scutellum); leaf (10 cm shoot stage); inflorescence 1 (young inflorescence at 5 mm); INF2: inflorescence 2 (inflorescences at 1–1.5 cm); root (from seedlings at 10 cm shoot stage); tiller (developing tillers at six leaf stage, 3rd internode); caryopsis 5 developing grain at 5 days post anthesis); caryopsis 15 (developing grain at 15 days post anthesis).

Tryptic digestion of proteins from a band of 95–110 kDa from an SDS-polyacrylamide gel of peak Id contained 37 peptides that matched the same α-glucosidase proteins, with a coverage of Agl97 of 47% (Fig 3A, S1 Table). Other proteins identified in the tryptic digests are listed in S2 Table and S3 Table.

Of the peptides that matched Agl1/Agl97, three (of seven, seven and six amino acids: Fig 3A) also matched the predicted product of a barley cDNA annotated as a GH31 α-glucosidase (BAK05419; http://www.cazy.org/GH31_eukaryota; [35]). This predicted protein is only 69% identical in amino acid sequence to Agl1/Agl97, and no other peptides from the tryptic digests matched it. All three matching peptides are also found in numerous other proteins with various functions from many species, and two [(R)VVAGYAK(A) and (R)QFLLGR(G)] are common to numerous α-glucosidases from microorganisms, animals and plants (information in CAZy GH31 family; www.cazy.org/GH31.html). There is thus no strong reason to believe that the product of BAK05419 was present in peak Id. Neither tryptic digest contained peptides that matched the predicted products of barley *Agl2* (GenBank: BAG72143; MLOC_57756, chromosome 3H), *Agl3* (GenBank: BAG72144; MLOC_77920.1, chromosome 1H) or *Agl4* (GenBank: BAG72145.1; MLOC_36871.1, chromosome 4H), identified by Tanaka et al. [24], apart from a seven amino-acid peptide common to Agl97, Agl4 and many other α-glucosidases [(R)RPFVLSR(S); S1 Table). The level of amino acid identity between Agls 2, 3 and 4 and Agl1/Agl97 is only 50% or less (Table 2). No other proteins predicted to be glucosidases were identified in the tryptic digests.

**Table 2:** Percentage of amino acid sequence identity between *Agl97*, *Agl1*, *Agl2*, *Agl3* and *Agl4*. Genbank accession numbers are shown.

	*Agl1*	*Agl97*	*Agl2*	*Agl3*	*Agl4*	Locus	Chromosome
Agl1_ACZ37245	100.0	99.0	50.1	32.5	31.1		7H
Agl97_AAF76254.1		100.0	49.9	32.4	30.9	MLOC_66806	7H
Agl2_BAG72143			100.0	30.6	30.4	MLOC_57756	3H
Agl3_BAG72144				100.0	30.8	MLOC_77920.1	1H
Agl4_BAG72145					100.0	MLOC_36871.1	4H

We found that both Agl97 and the longer predicted protein F2DV72 are encoded by a single gene (MLOC_66806) on the long arm of barley chromosome 7H. The same locus also encodes the α-glucosidase D1MDV2 (Agl1). The N-terminal peptide unique to the predicted protein F2DV72 is encoded by 159 nucleotides starting with an ATG translation initiation codon upstream of and in frame with the previously-identified *Agl1*/*Agl97* ATG codon (Fig 3B). Small sequence differences in clones obtained by different research groups (for example AAB02985.1 [16] and AAF76254.1 [18]) are almost certainly the result of use of different barley cultivars, rather than the existence of multiple, near-identical loci encoding Agl1/Agl97-like proteins.

Analysis of publicly-available data showed that *Agl1*/*Agl97* is expressed in several organs and developmental stages of the barley plant. Of the tissues tested, expression is strongest in the early stages of grain development, and in the embryo following germination (Fig 3C). *Agl2*, *Agl3* and *Agl4* are most highly expressed in the early stages of grain development. *Agl3* is also relatively highly expressed in the embryo following germination, although at a lower level than *Agl1*/*Agl97*.

## Discussion

Our results indicate that all of the maltase activity in the endosperm of barley seedlings is attributable to the product of a single gene. In line with previous studies, we found that maltase activity from seedling endosperm could be separated into distinct fractions with different charge properties. However, two lines of evidence indicate that the activity in all of the fractions is a function of the same or highly similar proteins. First, for a given amount of endosperm protein, maltase activity in all of the fractions was substantially higher in extracts of endosperm from wild-type plants than in extracts from two transgenic lines carrying an RNAi silencing cassette for *Agl97* [32]. This result implies that all of the maltase activity was a function of *Agl97* expression, or of expression of *Agl97* and other gene(s) very closely related in sequence to *Agl97* over the 411 bp region used in the RNAi construct.

Second, sequencing by mass spectrometry of proteins in the maltase-containing fraction from the MonoS column revealed the presence of the previously-characterised maltase Agl97. Tryptic peptides covered 47% of the predicted Agl97 protein. The same peptides are also encoded by *Agl1* and by a third sequence known from a cDNA AK367790.1. The products of *Agl97* and *Agl1* are identical in amino-acid sequence, and this protein has been shown experimentally to have maltase activity [19]. The predicted protein encoded by AK367790.1, F2DV72, is almost identical in sequence to Agl1/Agl97, apart from an N-terminal extension of 53 amino acids. A single, unique locus in the barley genome accounts for these sequences (MLOC_66806, chromosome 7).

The pattern of expression of Agl1/Agl97 is consistent with a role for the encoded maltase specifically during seed germination. In addition to publicly available transcript data showing a high level of expression in germinating grain at 4 dpi, transcript levels were shown by Northern blotting to increase strongly in germinating grains between 2 and 3 dpi–the point at which maltase activity appeared in our experiments–and to be elevated by treatment with gibberellin [16].

It seems likely that the multiple, separable maltase activities reported from barley endosperm in this and previous studies [20,22] are all derived by post-translational modification of Agl1/Agl97. Consistent with this assumption, Finnie et al. [36] reported that the Agl97 gene product appeared as five separate spots on 2-D gels of proteins secreted from isolated barley aleurone layers. We propose two possible explanations for the multiple activities/proteins derived from Agl1/Agl97. First, expression of the *Agl1*/*Agl97* gene may produce both a protein of 97 kDa and a 103 kDa protein with an additional N-terminal domain of 53 amino acids, using the two ATG codons in the first exon: both products would be expected to be processed to smaller sizes by removal of an N-terminal signal peptide upon export from the aleurone to the endosperm. The mature maltase protein of 92 kDa purified from endosperm by Frandsen et al. [18] may originate from the 97 kDa protein, which is predicted with confidence to have a cleavage site for an N-terminal signal peptide between amino acids 23 and 24. A transcript encoding the 103 kDa protein has been identified, but it is not known whether it is translated. Tibbot et al. [17] detected proteins estimated to be of 95 and 101 kDa in endosperm of germinating seeds using an antiserum raised to recombinant Agl97. However, the predicted 103 kDa protein has no apparent signal peptide cleavage site in the first 53 amino acids (SignalP 4.1, http://www.cbs.dtu.dk/services/SignalP-4.1) so it is not clear whether it could give rise to mature proteins of the masses detected by Tibbot et al.

Second, it seems likely that the product(s) of *Agl1*/*Agl97* is subject to extensive proteolysis following secretion from the aleurone into the endosperm. Several studies have reported the purification from barley endosperm of maltases with apparent masses considerably smaller than 97 kDa (e.g. [20,23]). Tibbot et al. [17] observed that the principal proteins in endosperm recognised by an Agl97 antiserum were of 101 and 95 kDa at 4 dpi, but of only 81 kDa two days later. Other possible modifications include variable levels of glycosylation and non-enzymic glycation, both of which have been proposed to account for the occurrence of multiple forms of other endosperm proteins [37,38].

There are at least four GH31 α-glucosidase-like proteins in addition to Agl1/Agl97 encoded in the barley genome ([24] and see http://www.cazy.org/GH31). These are less than 70% identical to Agl1/Agl97, and their substrate specificities are not known. Some of them may account for the peaks Ia, Ib and Ic of α-glucosidase activity that eluted from the MonoS column, since these fractions showed activity with the generic α-glucosidase substrate pNPG but not with maltose.

Our demonstration that the maltase activity of the barley endosperm is the product of a single gene opens the way for engineering this activity. For brewing purposes, it will be interesting to test whether maltase-free grain (obtainable by gene silencing, genome editing or mutant selection for the *Agl1*/*Agl97* gene) will give rise to lower glucose to maltose ratios in wort, and hence improved fermentation by yeast. Given that the maltase is apparently not required for normal germination and growth of barley [32], a maltase-free variety should be indistinguishable from normal barley in the field.

## Materials and Methods

### Plant material and grain germination

Grain of the UK elite barley variety NFC Tipple (2011 harvest) was a kind gift from Dr. Phil Howell (National Institute of Agricultural Botany, Cambridge, UK). Transgenic lines carrying an RNAi interference silencing cassette for *HvAgl97* (RNAi_*Agl97*_) were generated in cv Golden Promise [32]. Grains were surface sterilised according to Stanley et al. [32] and germinated on two filter papers (Whatman No. 1) moistened with 4 mL water in 9 cm petri dishes (10 grains per dish), at 17°C in the dark.

### Purification of α-glucosidases with maltase activity

Procedures were at 4°C unless otherwise stated. The roots and coleoptile of seedlings at 10 dpi were removed and the remaining tissue (referred to as the endosperm), was rapidly frozen and stored at -80°C until use. Tissue was ground under liquid nitrogen, homogenized in medium A [100 mM MOPS/KOH (pH 7.2), 1 mM EDTA, 1 mM DTT, 10% (v/v) ethanediol, with protease inhibitors (plant cocktail, Sigma-Aldrich, UK)], and filtered through two layers of muslin. After clarification by centrifugation, (20,000*g* for 30 min) ammonium sulfate was added to the filtrate and protein precipitating between 20 and 80% saturation was collected by centrifugation as above. The pellet was resuspended in medium B [20 mM HEPES/KOH (pH 7.5), 5 mM CaCl_2_, 5% (v/v) ethanediol], desalted with Sephadex G25 (PD-10 columns, GE Healthcare) equilibrated with the same medium, then applied to a MonoQ 5/50 GL anion exchange column (GE Healthcare) equilibrated with medium B, on an AKTA FPLC system (GE Healthcare). The column was washed with medium B at a flow rate of 1 mL min^-1^, then eluted with a 40-mL linear gradient of 0–1 M NaCl in medium B. Proteins in the initial wash (unbound MonoQ fraction) were desalted with Sephadex G25 equilibrated with medium C [25 mM Na acetate (pH 4.5), 5 mM CaCl_2_, 5% (v/v) ethanediol], then applied to a MonoS 5/50 GL cation exchange column equilibrated with medium C. The column was washed with medium C at a flow rate of 1 mL min^-1^, and eluted with a 40-mL linear gradient of 0–0.5 M NaCl in medium C.

### Mass spectrometric analysis and Mascot search parameters

Fractions collected during column chromatography were subjected to solid-phase extraction using OMIX C4 pipette tips (Agilent Technologies, UK), re-dissolved in 0.1 M triethylammonium bromide buffer (TEAB; Sigma-Aldrich), 0.1% (w/v) Rapigest (Waters Ltd, UK), reduced and alkylated with dithiothreitol and iodoacetamide respectively and digested with trypsin (Promega, UK) at 37°C for 16 h. Protein bands after SDS-PAGE were excised from colloidal Coomassie-stained gels and treated as described by Shevchenko et al. [39]. Peptides were extracted with 5% (v/v) formic acid, 50% (v/v) acetonitrile, dried down, and re-dissolved in 0.1% (v/v) TFA.

For LC-MS/MS analysis, samples were applied via a nanoAcquity^TM^ (Waters Ltd) UPLC^TM^-system at 250 nL min^-1^ to an LTQ-Orbitrap™ mass spectrometer (Thermo Fisher, MA), trapped with a Symmetry^®^ C18 pre-column (5 μm, 180 μm x 20 mm, Waters Ltd) in-line to an analytical BEH C18 column (1.7 μm, 75 μm x 250 mm, Waters Ltd) and eluted with a gradient of 3–37% (v/v. aqueous) acetonitrile, 0.1% (v/v) formic acid. The column was connected to a 10 μm SilicaTip™ nanospray emitter (New Objective, USA) attached to a nanospray interface (Proxeon, Denmark). MS was in positive ion mode at 200°C capillary temperature. The source and focusing voltages were tuned for the transmission of MRFA peptide (m/z 524) (Sigma-Aldrich, USA). Analysis was performed in Orbitrap-IT parallel mode (CID fragmentation of the five most abundant ions in each cycle, resolution of 30,000 over the MS range from m/z 350 to m/z 1800, MS target of 1xe6 and 1 s maximum scan time, collision energy 35, isolation width 2). Only mono-isotopic 2+ and 3+ charged precursors were selected for MS2 (minimal signal of 1000, AGC target of 3xe4 ions and 150 ms scan time, dynamic exclusion 1 count and 30 s exclusion, exclusion mass window ± 20 ppm).

Raw files were processed with MaxQuant 1.3.0.5 (http://maxquant.org) [40] to generate re-calibrated peaklist-files used for database searches. The searches were performed using Mascot® 2.3 Server (Matrix Science Limited, UK) on the SPtrEMBL fasta database (Sprot_sptrembl20120711) with taxonomy set to *Hordeum vulgare* (23,891 sequences) and a common contaminants database using trypsin/P with two missed cleavages and oxidation (M) and acetylation (N-term) as variable modifications. Mass tolerances were 6 ppm for precursor ions and 0.6 Da for fragment ions. Results were evaluated in Scaffold 3.6.4 (proteomsoftware.com, Portland, USA) with thresholds of 99% and 95% for proteins and peptides respectively, resulting in a false discovery rate of 0% for both peptides and proteins.

### Enzyme assays

Endosperm extracts were prepared as for enzyme purification. Samples were desalted with Sephadex G25 (coarse) columns equilibrated with 50 mM sodium acetate (pH 5). α-Glucosidase and maltase were assayed with pNPG and maltose respectively as described by Stanley et al. [32].

## Supporting Information

S1 Table Maltase-related peptides found in peak Id following MonoS purification. (DOCX)

S2 Table Proteins identified in solution from peak 1d. (DOCX)

S3 Table Proteins identified following SDS-PAGE of peak 1d. (DOCX)
