# Supplementary material for: The Maltase Involved in Starch Metabolism in Barley Endosperm Is Encoded by a Single Gene
Source: PLoS One. 2016 Mar 24;11(3):e0151642. doi: 10.1371/journal.pone.0151642 (PMC4807107; doi:10.1371/journal.pone.0151642)
Supplement: S1 Table — (DOCX) [file pone.0151642.s001.docx]

**S1 Table** Maltase-related peptides found in peak Id following MonoS purification

| Identification from proteins in solution | | | | | |
| --- | --- | --- | --- | --- | --- |
| Matching proteins from database | | | Coverage (%) | Unique peptides | Peptide sequences^a^ |
| Protein | | Accession |  |  |  |
| High pI α-glucosidase^b^  (Agl97)  Predicted protein  α-glucosidase  (Agl1) | Q9LLY2  F2DV72  D1MDV2 | | 22 | 13 | - (R)AEAAAATGGASSTGDVQR(L) - (R)VLSAAGSDLVLTVHASPFR(F) - (R)STGDILFDTAPGLVFR(D) - (K)YLEVTSALPAGR(A) - (R)ASLYGLGEHTK(S) - (R)IDATYGTFVR(G) - (R)GMQQDIFLK(R) - (R)YTAHWTGDNAATWGDLR(Y) - (R)WIQLGAFYPFSR(D) - (R)PLFFSYPHDVATYGVDR(Q) - (K)HVTLPAPADTVNVHVAGGTILPL QQSALTTSR(A) - (R)FScEMGSDGAIK(V) - (K)SEVVHNSYAQSR(T) |
| Identification after SDS-PAGE, band size 95-110 kDa | | | | | |
| Matching proteins from database | | | Coverage (%) | Unique peptides | Peptide sequences^a^ |
| Protein | | Accession |  |  |  |
| High pI α-glucosidase  (Agl97)  α-glucosidase  (Agl1) | | Q9LLY2  D1MDV2 | 47 | 37 | - (R)VAVDDGGR(R) - (R)LRAEAAAATGGASSTGDVQR(L) - (R)AEAAAATGGASSTGDVQR(L) - (R)LAVYASLETDSR(L) - (R)VRITDADHPR(W) - (R)WEVPQDIIPR(P) - (R)PAPADVLHDAPPASSAPLQGSR(V) - (R)VLSAAGSDLVLTVHASPFR(F) - (R)STGDILFDTAPGLVFR(D) - (R)DKYLEVTSALPAGR(A) - (K)YLEVTSALPAGR(A) - (R)ASLYGLGEHTK(S) - (R)PAPmPYWSFGFHQcR(Y) - (R)VVAGYAK(A) - (K)YVLILDPGIR(I) - (R)IDATYGTFVR(G) - (R)GMQQDIFLK(R) - (R)GMQQDIFLKR(N) - (R)AAEFWAR(E) - (K)TVPASAVHYGGVTEYDAHNLFGLLEAR(A) - (R)RPFVLSR(S) - (R)STFVGSGR(Y) - (R)YTAHWTGDNAATWGDLR(Y) - (R)WIQLGAFYPFSR(D) - (R)DHSAIFTVR(R) - (R)ELYLWPSVAASAR(K) - (R)PLFFSYPHDVATYGVDR(Q) - (R)QFLLGR(G) - (R)GVLVSPVLEPGATTVDAYFPAGR(W) - (R)WYSLYDYSLAVATR(T) - (R)SDWSMVR(F) - (R)FS(c)EM(o)GSDGAIK(V) - (R)FS(c)EMGSDGAIK(V) - (K)VKSEVVHNSYAQSR(T) - (K)SEVVHNSYAQSR(T) - (K)VVLMGHR(S) - (K)KLTVHVNSAEVEASSSAGTR(Y) |

Identification of peptides was accepted if they could be established at >95.0% probability by the Peptide Prophet algorithm (Keller et al., 2002). Protein identifications were accepted if they could be established at a >99.0% probability threshold and contained at least 1 identified peptide.

^a^Lower case letter in brackets indicates modifications of the preceding amino acid. c: carbamidomethyl; o: oxidation

^b^Proteins that contained similar peptides and could not be differentiated based on MS/MS analysis alone were grouped to satisfy the principles of parsimony.

The three highlighted peptides are common to Agl1/Agl97 and a predicted α-glucosidase BAK05419. The peptide highlighted in yellow is common to these two proteins plus Agl4 (BAG72145).

Keller A, Nesvizhskii AI, Kolker E, Aebersold R. Empirical statistical model to estimate the accuracy of peptide identifications made by MS/MS and database search. Anal. Chem. 2002; 74: 5383-5392.
