# Supplementary material for: The Maltase Involved in Starch Metabolism in Barley Endosperm Is Encoded by a Single Gene
Source: PLoS One. 2016 Mar 24;11(3):e0151642. doi: 10.1371/journal.pone.0151642 (PMC4807107; doi:10.1371/journal.pone.0151642)
Supplement: S2 Table — (DOCX) [file pone.0151642.s002.docx]

**S2 Table** Proteins identified in solution from peak 1d

Protein identifications were accepted if they could be established at a >99.0% probability threshold and contained at least 1 identified peptide. The % coverage by peptides of the predicted sequence and the number of peptides identified are given.

| **Identified Proteins** | **Accession number** | **Mol. Wt (kDa)** | **Coverage (%)** | **Unique peptides** |
| --- | --- | --- | --- | --- |
| Beta-glucosidase | B5A496 | 57 | 52.1 | 21 |
| Alpha-amylase | C3W8N0; P04063 | 47 | 63.9 | 20 |
| Protein synthesis inhibitor I; Ribosome-inactivating protein I / rRNA N-glycosidase | P22244 | 30 | 48 | 11 |
| Beta-amylase | Q84T20; Q9AVJ8; Q9FUK6 | 60 | 47.3 | 20 |
| 26 kDa endochitinase 1 | P11955 | 33 | 52.5 | 13 |
| Beta-galactosidase; | F2EF11 | 79 | 8.1 | 4 |
| Xylanase inhibitor | Q6KE44 | 41 | 37 | 13 |
| 60 kDa jasmonate-induced protein; rRNA N-glycosidase | Q00531 | 60 | 12.3 | 5 |
| Alpha-glucosidase | Q9S973; D1MDV2; F2DV72 | 97 | 22.4 | 13 |
| Non-specific lipid-transfer protein | A8YPK3; F2ED95; P07597 | 12 | 65 | 5 |
| Isocitrate lyase | F2CWQ8 | 63 | 21.6 | 11 |
| Alpha-galactosidase; | O04944 | 22 | 43.1 | 8 |
| Lichenase-2; Endo-beta-1,3-1,4 glucanase II | P12257 | 33 | 23.7 | 5 |
| Ascorbate peroxidase | O23983 | 27 | 33.6 | 7 |
| 26 kDa endochitinase 2 | P23951 | 28 | 36.5 | 4 |
| Aminomethyltransferase | F2DZ70 | 44 | 13.6 | 4 |
| Gamma-hordein-3 | P80198 | 33 | 9.69 | 3 |
| Serpin-Z7; BSZ7; HorvuZ7 | Q43492 | 43 | 8.06 | 3 |
| Hordoindoline b-2 | G1UH43; Q5IUA2; Q9LEH8 | 16 | 14.3 | 2 |
| Probable non-specific lipid-transfer protein; LTP; Aleurone-specific 10 kDa protein; B-FABP | P20145 | 10 | 24.5 | 2 |
| Alpha-amylase inhibitor BMAI-1; Alpha-amylase flour inhibitor; Allergen Hor v 1 | P16968 | 16 | 17.1 | 2 |
| Non-specific lipid-transfer protein | Q5UNP2 | 12 | 18.5 | 2 |
| Alpha-amylase | C3W8M9 | 48 | 36.5 | 2 |
| Non-specific lipid-transfer protein | F2CY84; Q9SES6 | 12 | 28.1 | 2 |
| Serpin-Z4; BSZ4; HorvuZ4 | P06293 | 43 | 5.01 | 2 |
