# Supplementary material for: The Maltase Involved in Starch Metabolism in Barley Endosperm Is Encoded by a Single Gene
Source: PLoS One. 2016 Mar 24;11(3):e0151642. doi: 10.1371/journal.pone.0151642 (PMC4807107; doi:10.1371/journal.pone.0151642)
Supplement: S3 Table — (DOCX) [file pone.0151642.s003.docx]

**S3 Table** Proteins identified following SDS-PAGE of peak Id

Protein identifications were accepted if they could be established at a >99.0% probability threshold and contained at least 1 identified peptide. The % coverage by peptides of the predicted sequence and the number of peptides identified are given.

(a) and (b) are from bands of 95-100 kDa and ~110 kDa, respectively.

(a)

| **Identified Proteins** | **Accession number** | **Coverage (%)** | **Unique peptides** |
| --- | --- | --- | --- |
| Protein synthesis inhibitor I; Ribosome-inactivating protein I; rRNA N-glycosidase | P22244 | 33.1 | 8 |
| Alpha-amylase | C3W8N0 | 37.9 | 14 |
| Lipoxygenase | F2CWD2 | 26.7 | 23 |
| Beta-glucosidase | B5A496 | 54 | 25 |
| Lichenase-2; Endo-beta-1,3-1,4 glucanase II | P12257 | 26 | 6 |
| Xylanase inhibitor | Q6KE44 | 20.6 | 6 |
| Alpha-glucosidase | Q9LLY2; D1MDV2 | 45.1 | 36 |
| Beta-amylase | Q84T20; Q9FUK6; Q9AVJ8 | 41.7 | 18 |
| Non-specific lipid-transfer protein | A8YPK3; P07597; F2ED95 | 23.9 | 2 |
| Alpha-galactosidase | O04944 | 30.4 | 6 |
| Peroxidase | O49866 | 12.6 | 4 |

(b)

| Protein synthesis inhibitor I; Ribosome-inactivating protein I; rRNA N-glycosidase | P22244 | 38.8 | 8 |
| --- | --- | --- | --- |
| Alpha-amylase | C3W8N0 | 61.4 | 20 |
| Lipoxygenase | F2CWD2 | 69.1 | 58 |
| Beta-glucosidase | B5A496 | 63.5 | 26 |
| Lichenase-2; Endo-beta-1,3-1,4 glucanase II | P12257 | 31.1 | 6 |
| Xylanase inhibitor | Q6KE44 | 37.5 | 11 |
| Beta-galactosidase | F2EF11 | 11.3 | 4 |
| Alpha-glucosidase | Q9LLY2; D1MDV2 | 36.5 | 24 |
| Beta-amylase | Q84T20; Q9FUK6; Q9AVJ8 | 43.6 | 17 |
| Beta-D-glucan exohydrolase, isoenzyme ExoII | Q42835 | 11.2 | 7 |
| Non-specific lipid-transfer protein | A8YPK3; P07597; F2ED95 | 38.5 | 3 |
| Alpha-galactosidase | O04944 | 44.6 | 9 |
| Isocitrate lyase | F2CWQ8 | 7.5 | 3 |
| Beta-D-xylosidase | Q8W011 | 2.7 | 2 |
| Endochitinase 2 | P23951 | 25.6 | 2 |
| Jasmonate induced protein | Q43490 | 9.24 | 4 |
| 60kDa jasmonate-induced protein; rRNA N-glycosidase | Q00531; Q9S973 | 12.9 | 4 |
| Lipoxygenase | Q42847 | 67.9 | 3 |
| Purple acid phosphatase isoform a | C4PKL2; F2DF56 | 5.15 | 2 |
